# Supplementary material for: Content-rich biological network constructed by mining PubMed abstracts
Source: BMC Bioinformatics. 2004 Oct 8;5:147. doi: 10.1186/1471-2105-5-147 (PMC528731; doi:10.1186/1471-2105-5-147)
Supplement: Additional File 2 — The original results of the above study (non-essential files are deleted to keep the file size under the limit set by BMC bioinformatics). [file 1471-2105-5-147-S2.bz2 › chilibotAdditionalFile2/dip10/331ID1328224E3927/html/TNFRSF1B_LTA.html]

 


 **TNFRSF1B** and **LTA** 
  
Found 38 abstracts in PubMed, retrieved 10.  
 

 What does Google say? 
 PDF only 
| .edu only 

---

**Interactive relationship** (e.g. stimulation, inhibition, etc)

**Stimulatory relationship**- In vitro, constitutive and stimulated production of TNF beta  [ **LTA** ] , IL 1beta, MIP 1beta, and IL 8 by normal mononuclear cells declined with etanercept  [ **TNFRSF1B** ] , detectable TNF alpha increased.  Ref: 11458980 Res Commun Mol Pathol Pharmacol, 2001
**Neutral relationship**- Composed of 2 soluble TNF receptor p75 domains fused to human immunoglobulin, etanercept  [ **TNFRSF1B** ]  neutralizes the inflammatory cytokines TNF and lymphotoxin alpha  [ **LTA** ] .  Ref: 12463460 Clin Exp Rheumatol< MedlineTA>Clin Exp Rheumatol, 2001
- LT consists of soluble LT alpha  [ **LTA** ]  3, which binds to TNF R1 and TNF R2  [ **TNFRSF1B** ] , and membrane LT alpha  [ **LTA** ]  1 beta 2, which binds to LT beta R.  Ref: 12882833 Int Immunol, 2003

**Non-interactive relationship** (e.g. studied together, co-existance, homology, etc.)

- This activity of LT depends on membrane LT alpha  [ **LTA** ]  1 beta 2 and not soluble LT alpha  [ **LTA** ]  3, since DA virus persisted and induced inflammatory demyelination in LT beta R, but not TNF R1 or TNF R2  [ **TNFRSF1B** ] , mice.  Ref: 12882833 Int Immunol, 2003
- Use of gene knockout mice indicates that depletion of BM B cells is dependent on TNF alpha, lymphotoxin alpha  [ **LTA** ] , and both TNF receptors, TNFR1 p55 and TNFR2  [ **TNFRSF1B** ]  p75.  Ref: 12444124 J Immunol, 2002
- We investigated the effect of a TNF and LT alpha  [ **LTA** ]  double deficiency on sleep in mice Ligand KO and compared their sleep with that of R1KO, TNFR2  [ **TNFRSF1B** ]  knockout R2KO mice, and wild type WT controls.  Ref: 12163535 J Neurophysiol, 2002
